# Effects of Crystallite Sizes of Pt/HZSM-5 Zeolite Catalysts on the Hydrodeoxygenation of Guaiacol

**DOI:** 10.3390/nano10112246

**Published:** 2020-11-12

**Authors:** Haonan Duan, Yajie Tian, Siyuan Gong, Bofeng Zhang, Zongjing Lu, Yinqiang Xia, Yawei Shi, Congzhen Qiao

**Affiliations:** 1 Henan Province Engineering Research Center of Catalytic Reaction, College of Chemistry and Chemical Engineering, Henan University, Kaifeng 475004, China; dhn377143955@163.com; 2 Key Laboratory for Green Chemical Technology of Ministry of Education, School of Chemical Engineering and Technology, Tianjin University, Tianjin 300072, China; gsygsy@tju.edu.cn (S.G.); zhangbofeng@tju.edu.cn (B.Z.); zongjinglu@tju.edu.cn (Z.L.); 3 College of Food Science and Engineering, Northwest A&F University, Yangling 712100, China; 4 College of Environmental Science and Engineering, Dalian Maritime University, Dalian 116026, China; shiyawei@tju.edu.cn

**Keywords:** hydrodeoxygenation, guaiacol, Pt/HZSM-5 catalyst, hierarchical zeolite, crystallite size

## Abstract

Herein, Pt/HZSM-5 zeolite catalysts with different crystallite sizes ranging from nanosheet (~2 nm) to bulk crystals (~1.5 μm) have been prepared for the hydrodeoxygenation of guaiacol, and their effects on the reaction pathway and product selectivity were explored. HZSM-5 zeolites prepared by seeding (Pt/Z-40: ~40 nm) or templating (Pt/NS-2: ~2 nm) fabricated intra-crystalline mesopores and thus enhanced the reaction rate by promoting the diffusion of various molecules, especially the bulky ones such as guaiacol and 2-methoxycyclohexanol, leading to a higher cyclohexane selectivity of up to 80 wt % (both for Pt/Z-40 and Pt/NS-2) compared to 70 wt % for bulky HZSM-5 (Pt/CZ: ~1.5 μm) at 250 °C and 120 min. Furthermore, decreased crystallite sizes more effectively promoted the dispersion of Pt particles than bulky HZSM-5 (Pt/Z-400: ~400 nm and Pt/CZ). The relatively low distance between Pt and acidic sites on the Pt/Z-40 catalyst enhanced the metal/support interaction and induced the reaction between the guaiacol molecules adsorbed on the acidic sites and the metal-activated hydrogen species, which was found more favorable for deoxygenation than for hydrogenation of oxygen-containing molecules. In addition, Pt/NS-2 catalyst with a highly exposed surface facilitated more diverse reaction pathways such as alkyl transfer and isomerization.

## 1. Introduction

Owing to the increasing air pollution levels and decreasing supplies of fossil fuels, bio-oil is considered a promising chemical raw material and liquid fuel. [1,2,3]. However, the bio-oils produced by liquefaction or fast pyrolysis have very high oxygen contents (up to 50 wt %), which not only decrease energy density to a value lower than that of petroleum fuel by a factor of two (17 kJ/kg versus 40 kJ/kg), but also result in high viscosity, low pH values, and poor chemical stability. For these reasons, bio-oil cannot be blended with petroleum distillates or used directly in engines [4,5]. Hydrodeoxygenation (HDO), catalytic pyrolysis, and steam reforming are commonly used techniques for bio-oil upgrading, among which HDO is the most widely studied and promising method for increasing the H/C ratio and decreasing the O/C magnitude [6,7].

Phenolic compounds (including phenol and its substituted derivatives guaiacol and syringol) are often selected as model compounds for the primary bio-oil upgrading, which account for almost one fourth of the oxygen-containing bio-oil components [8]. Under various catalytic conditions of the HDO process, phenolic compounds are transformed to different products (including benzene, cyclic alcohols, and cycloalkanes) through the hydrogenation, alkyl transfer, demethoxylation, and dehydration reactions. For example, the catalytic HDO of guaiacol (containing both sp2 Caryl–O and sp3 CH_3_–O bonds) into cyclohexane, a fully deoxygenated product with a high calorific value and low oxygen content, has been widely studied for bio-oil upgrading [9,10].

Traditional hydrodesulfurization and hydrodenitrification catalysts such as sulfide-based NiMo/γ-Al_2_O_3_ and CoMo/γ-Al_2_O_3_ have been also considered for possible HDO applications; however, their use resulted in high degrees of product pollution with leaching of sulfide species [11]. Currently, an increasing number of research groups are investigating environmentally friendly sulfur-free HDO catalysts. Transition metal catalysts such as metal sulfides, nitrides, and carbides exhibit relatively high deoxygenation activities [12]. Recently, bifunctional metal–solid acid HDO catalysts have attracted much attention from researchers, owing to the presence of active metal sites that promote C=C/C=O hydrogenation and acidic sites for the deoxidation of aliphatic oxygenates. Among these compounds, the catalysts containing noble metals (such as Pt, Pd, or Ru) demonstrated excellent performance characteristics at lower hydrogen pressures than those required for the direct hydrogenolysis over metal nanoparticles alone [13].

Zeolites with moderate acidities and regular pore structures were found to be more suitable substrates for phenol hydrodeoxygenation catalysts than silica/alumina carriers. Moreover, zeolites with medium pore sizes such as acidic ZSM-5 (HZSM-5) exhibited relatively high catalytic activities [14,15]. Apart from the high hydrothermal stability and medium acidity of HZSM-5, its advanced crystal network containing both sinusoidal (0.51 × 0.55 nm) and interconnecting straight (0.56 × 0.53 nm) channels allows easy access of bio-liquid components and achieving a desired product shape selectivity [16,17]. For example, Grunwaldt et al. obtained a cyclohexane yield higher than 90% during the HDO of guaiacol using Pt/HZSM-5 (Si/Al = 90) catalyst at a reaction temperature of 200 °C [18]. Unfortunately, bifunctional zeolite-supported noble metals also have several disadvantages. The metal aggregation on the carrier surface during the high-temperature oxidation–reduction and limited mass transport of guest molecules inside the micropores significantly decrease their catalytic activity and cause fast catalyst deactivation, especially in the case of bulky oxygenated compounds [19,20].

The development of hierarchical zeolites containing secondary macroporous or mesoporous networks is considered an efficient way of facilitating the reactant diffusion and enhancing the catalyst performance [21,22]. The current synthetic strategy for hierarchical zeolites includes shortening the diffusion path length via the preparation of nanocrystalline zeolites and generation of mesopores within zeolite crystals [23]. The hierarchical pore structure promotes the reactant mass transfer and binding of intermediate products to the catalyst active sites; it also increases the reaction rate and conversion. Moreover, the high surface area of such a structure facilitates the dispersion of metal particles and utilization of metal active sites [24]. For example, Pt catalyst supported on mesoporous HZSM-5 was more active during the HDO of dibenzofuran and 2-methoxy phenol than its conventional microporous zeolite counterparts [25]. Wilson and co-workers [26] found that the hierarchical Pd/HZSM-5 catalyst contained highly dispersed palladium nanoparticles characterized by high activity and selectivity for the HDO conversion of m-cresol to methylcyclohexane via stepwise hydrogenation and deoxygenation combined with enhanced mass transport properties.

Seed-induced secondary growth is a common method for controlling zeolite crystal sizes and generating intra-crystalline mesopores or macropores [27]. The addition of zeolite seeds can significantly increase the nucleation rate and allows adjusting the grain size distribution in the range from several micrometers to several nanometers. After decreasing the size of zeolite crystals, the surface area of zeolite catalysts increases significantly, and the formation of intra-crystalline mesopores accelerates the diffusion process, enhancing the catalytic performance (especially in the case of bulky reactants) [28]. Meanwhile, the successful synthesis of 2-dimensional lamellar ZSM-5 catalyst using a bi-quaternary ammonium surfactant represents a new route for the preparation of hierarchical ZSM-5 zeolite containing stacks of zeolite nanosheets with relatively short diffusion paths (approximately 2 nm) across the b-axis [29]. The catalytic performance on this spatial scale is considerably improved by facilitating the transport of reactants towards the acidic sites on the nanosheet surface [30]. Tsapatsis et al. observed more efficient adsorption of n-nonane, n-hexane, and i-hexane on hierarchical zeolite nanosheets as compared with that on the microporous zeolite surface, owing to the adsorbate condensation in their mesopores [31].

Thus, the noble metal supported on hierarchical HZSM-5 zeolite catalysts with different crystallite sizes would exhibit enhanced catalytic activity and product selectivity during the HDO of phenolic substances due to their superior diffusion properties and distribution of acidic sites. Therefore, it is highly desirable to examine various steps of this process (including the hydrogenation, demethoxylation, and dehydration ones), leading to the cleavage of C–O bonds in phenol derivatives over the catalysts with different zeolite crystal sizes. As of today, very few studies related to the HDO of bio-oil phenolic substances have been performed. In this work, HZSM-5 zeolites with different crystal sizes (40 and 400 nm) were synthesized from silicalite-1 seeds by the secondary growth method. Nanosheet HZSM-5 (NS-Z5) zeolite was produced using C_22_H_45_N(CH_3_)_2_C_6_H_12_N(CH_3_)_2_C_6_H_13_Br_2_ as a structure-directing agent (SDA). Commercial ZSM-5 zeolite with a crystal size of approximately 1.5 μm was utilised as a reference. Pt-loaded HZSM-5 catalysts with a theoretical loading of 1 wt % were prepared by incipient wetness impregnation. Several characterization methods, including scanning electron microscopy (SEM, Hitachi, Tokyo, Japan), transmission electron microscopy (TEM, JEOL, Tokyo, Japan), hydrogen temperature-programmed reduction (H_2_–TPR, Micromeritics, Pittsburgh, PA, USA), X-ray diffraction (XRD, Philips, Amsterdam, The Netherlands), X-ray photoelectron spectroscopy (XPS, Thermo Scientific, Waltham, MA, USA), ammonia temperature-programmed desorption (NH_3_–TPD, Micromeritics, Pittsburgh, PA, USA), CO chemisorption (Micromeritics, Pittsburgh, PA, USA), and N_2_ physisorption (Micromeritics, Pittsburgh, PA, USA) were used to determine the morphology, pore structure, metal particle distribution, and acidic properties of the prepared catalysts. The HDO of guaiacol was performed inside a high-pressure batch reactor. From the obtained product distributions, the effects of the HZSM-5 zeolite carrier size on the parameters of the phenolic bio-oil HDO process and product selectivity were investigated. Finally, the conversion rates of guaiacol and its intermediates were determined to illustrate the difference between the aliphatic and aromatic C–O bonds in the oxygen-containing hydrocarbons.

## 2. Materials and Methods

### 2.1. Catalyst Preparation

NS-Z5 catalyst was synthesized using [C_22_H_45_N(CH_3_)_2_C_6_H_12_N(CH_3_)_2_C_6_H_13_]Br_2_ as an SDA [32]. Bulky HZSM-5 zeolite with a crystallite size along the long axis (Z5) of approximately 400 nm was prepared in the presence of tetrapropylammonium hydroxide (TPAOH). Details of the utilised preparation method are provided in the Supporting Information section.

Nanocrystalline MFI zeolite with a crystallite size of 40 nm was synthesized by the seeding technique. Silicalite-1 seeds with dimensions of approximately 60 nm were prepared via the following procedure (their characteristics are presented in the Figure S1) [33]. First, a reaction solution (molar ratio of 1 TPAOH: 2.8 TEOS: 40 H_2_O) was aged at 25 °C for 24 h under stirring. After that, it was transferred into a Teflon-lined autoclave and stirred at a rotation speed of 30 rpm and temperature of 80 °C for 72 h. The obtained crystals were washed by deionized water until the pH of the resulting suspension became close to 7–8 followed by calcination at 550 °C for 4 h. A desired amount of silicalite-1 seeds was added to the gel synthesized by the Z5 preparation method [28]. The proportion of silicalite-1 seeds in the total silicon amount was 1 wt %, and the obtained product was labelled Z-40. Commercial HZSM-5 zeolite with a long axial length of 1.5 μm was purchased from Nankai University (Tianjin, China) and labelled CZ.

Protonated HZSM-5 zeolite was prepared through the ion exchange with 1 mol/L NH_4_NO_3_ solution. The obtained product was loaded with 1 wt % platinum metal from H_2_PtCl_6_ solution via incipient wetness impregnation. After sequential drying at 30, 60 and 120 °C for 12 h, the produced zeolite samples were calcinated at 500 °C for 4 h. The produced zeolite samples with different zeolite crystal sizes were labelled as Pt/Z-400, Pt/Z-40, and Pt/NS-2, corresponding to zeolite with thickness of 400 (bulky), 40 (nano-sized) and 2 nm (nanosheet layers). Pt loaded on commercial ZSM-5 zeolite was labelled as Pt/CZ.

### 2.2. Characterization Methods

The surface morphology of zeolites was observed through a scanning electron microscopy (SEM, Hitachi, Tokyo, Japan). Transmission electron microscope (TEM) images were collected on a JEM-2100F (JOEL, Tokyo, Japan) microscope. Elemental analysis measurements were performed on an inductively coupled plasma (ICP) spectrophotometer (ICP-9000(N+M), Thermo Jarrell-Ash Corp, Waltham, USA). X-ray diffraction (XRD) patterns were recorded on a Philips X’Pert MPD diffractometer (Philips, Amsterdam, Netherlands) equipped with Cu-Kα radiation. Nitrogen adsorption-desorption isotherms were measured at −196 °C using a ASAP 2420 volumetric adsorption analyzer (Micomeritics, Pittsburgh, USA). Ammonia temperature-programmed desorption (NH_3_-TPD) was performed on an AMI-300 (Altamira Instruments, Pittsburgh, USA). Typically, 100 mg of samples were used for each measurement. The sample was pretreated at 500 °C in the Helium gas flow for 30 min to eliminate impurities. After cooling to 50 °C, the sample was saturated with 5% NH_3_/He and the gaseous and weakly adsorbed NH_3_ were subsequently removed by purge with He for 60 min. The temperature was raised to 550 °C at a rate of 10 °C/min and the NH_3_-TPD profile was recorded by a TCD detector. X-ray photoelectron spectra (XPS) was conducted on a Thermo ESCALAB 250XI instrument (Thermo Scientific, Waltham, USA). The binding energy (BE) results were calibrated by turning that of C1s peak to 285.0 eV. Samples before detection were reduced under H_2_(10%)/Ar(90%) atmosphere at 400 °C and then decreased to room temperature under N_2_ atmosphere. To maximum prevent the oxidation, the prepared samples for XPS detection was put in sealing tube filled with N_2_. CO-chemisorption was performed on the Chemisorption Analyzer (AMI-300, Altamira Instruments, Pittsburgh, USA). Before the test, the catalysts were reduced in situ at 450 °C for 2 h in flow of hydrogen (30 mL/min), then purged with He (30 mL/min) for 40 min. The samples were cooled to 35 °C and then 15 pulses of 37.5% CO/62.5% He were injected at regular intervals. The Pt dispersion and average Pt particle size were calculated by assuming an adsorption of one CO molecule per Pt atom. Temperature programmed reduction (TPR) information was determined on a conventional device equipped with a thermal conductivity detector (AutoChem1 II 2920, Micromeritics, Pittsburgh, USA). The 50 mg sample was pretreated to 400 °C for 30 min at a rate of 10 °C/min in He flow to eliminate adsorbed water. After cooled down to room temperature, 25% H_2_/N_2_ at 60 mL/min was used as the reduction gas mixture, and the temperature was raised from room temperature to 700 °C with the heating rate was 10 °C/min.

### 2.3. Catalyst Evaluation

First, all catalysts were reduced at 400 °C in the presence of a flowing H_2_ gas (10 vol.% in N_2_, 50 mL/min) for 4 h. HDO of guaiacol was performed in a 100-mL stainless steel batch autoclave. For a single run, 0.5 g of guaiacol, 25 mL of n-dodecane, and 0.1 g of a studied catalyst were placed inside the reactor. The initial reaction pressure of hydrogen gas was maintained at 4 MPa after displacing the air with H_2_ five times. Subsequently, the reaction temperature was increased to 250 °C. After a specified reaction time, the autoclave was immediately cooled in an ice-water bath, and the obtained products were analyzed by a gas chromatograph (GC-7980, Techcomp, Beijing, China) equipped with a flame ionization detector and an HP-5 column (inner diameter: 0.32 mm, length: 50 m). The product distribution as a function of time was determined by conducting separate batch reactions with different durations.

## 3. Results

### 3.1. Catalyst Properties

**Figure 1:**
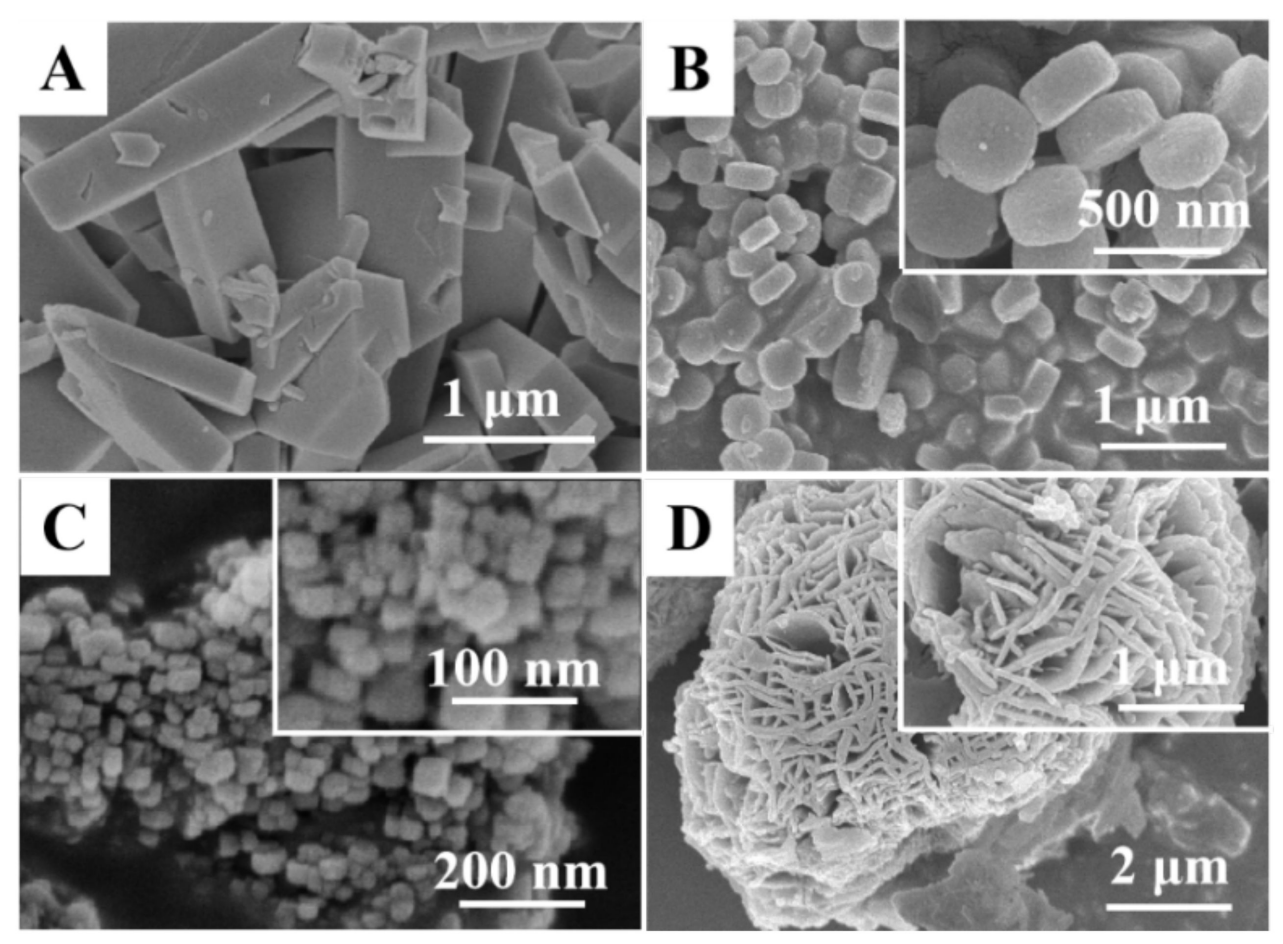
SEM images of: (**A**) Pt/CZ, (**B**) Pt/Z-400, (**C**) Pt/Z-40, and (**D**) Pt/NS-2 catalysts.

Figure 1 displays the SEM images of the Pt-loaded catalysts with different morphologies. The commercial Pt/CZ catalyst exhibits coffin-shaped particles with sizes of approximately 1.5 μm along the long axis direction (Figure 1A). The crystallite size of HZSM-5 zeolite prepared using TPAOH (Pt/Z-400 in Figure 1B) is considerably lower than 1.5 μm (approximately 400 nm). After adding 1 wt % silicalite-1 seeds, nanocrystals with a size of approximately 40 nm formed 200–1000 nm aggregates (Figure 1C). It is commonly assumed that nanocrystal seeds first decompose into small crystal nuclei, after which silicon and aluminum sources aggregate around these nuclei into zeolite crystals through Oswald ripening. This process is promoted by the seed addition to the synthesized solution that decreases the crystal size [23]. Finally, the nanosheet ZSM-5 zeolite depicted in Figure 1D exhibits a flower-like morphology composed of curved layers, which is consistent with the results of previous studies [34]. Some macropores were located between zeolite sheets.

**Figure 2:**
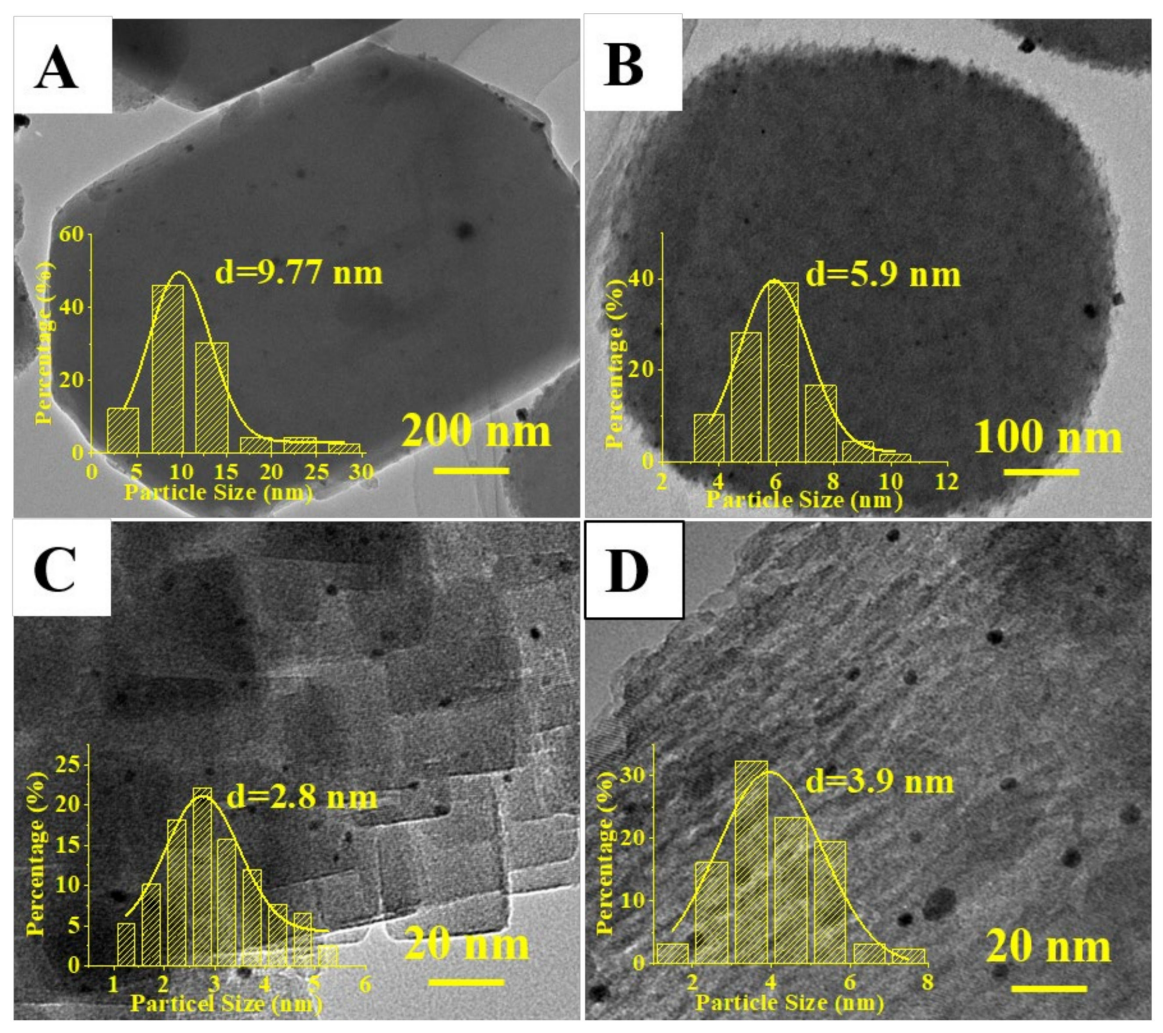
TEM images of (**A**) Pt/CZ, (**B**) Pt/Z-400, (**C**) Pt/Z-40, and (**D**) Pt/NS-2 catalysts.

The catalyst supports and Pt particle distributions were also characterised by TEM (Figure 2). Here, coffin-shaped structures were detected for the Pt/CZ (Figure 2A) and Pt/Z-400 (Figure 2B) samples. Pt/Z-40 catalyst was composed of nano-crystals with dimensions of approximately 40 nm (Figure 2C). Pt/NS-2 catalyst contained stacks of several nanosheet layers (Figure 2D, thicknesses of approximately 2.1 nm in the as-synthesized zeolite, not shown). The diameter of Pt nanoparticles also varied with the dimensions of the zeolite support prepared by incipient wetness impregnation. The Pt nanoparticle size decreased with decreasing support size in the order Pt/CZ (9.7 nm) > Pt/Z-400 (5.9 nm) > Pt/Z-40 (2.8 nm), as shown in Table 1. For the HZSM-5 nanosheet-supported Pt sample, the Pt particle size measured by TEM was 3.9 nm, which was larger than that of Pt/Z-40 catalyst. This phenomenon can be attributed to the metal agglomeration on the support with a high surface area after the sequential calcination and reduction steps.

**Figure 3:**
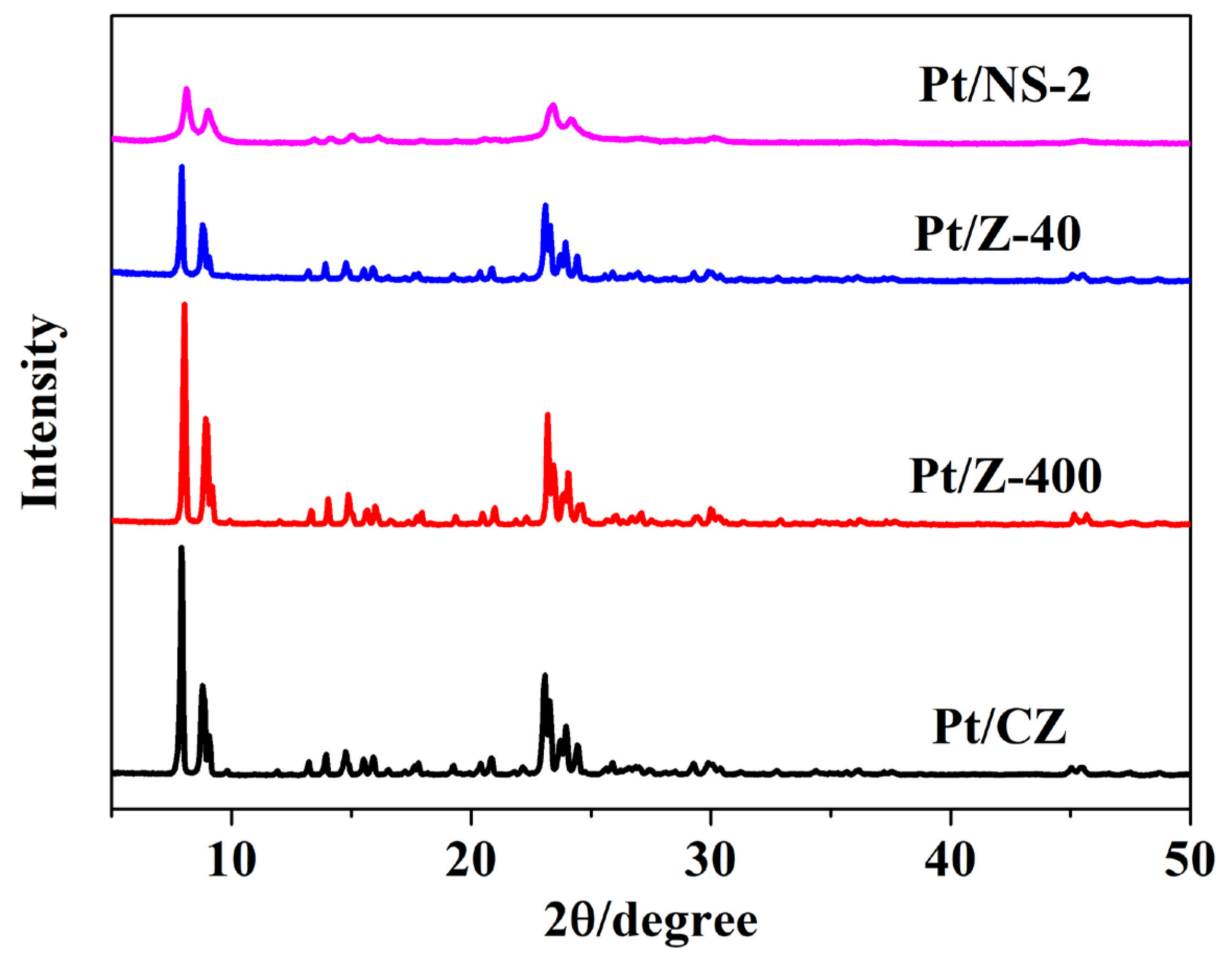
XRD patterns of the prepared Pt-loaded zeolites.

Figure 3 shows the XRD patterns of the Pt-loaded catalysts. All the studied samples contain the MFI crystal phase according to the standard PDF card generated by Jade 6 software (JCPDS-46-0003). Moreover, its peak intensity decreased in the order Pt/CZ > Pt/Z-400 > Pt/Z-40 > Pt/NS-2, which was consistent with the lower crystal sizes. No Pt diffraction peak was detected due to either the presence of highly dispersed metal sites or the very low Pt content that was below the XRD detection limit.

**Figure 4:**
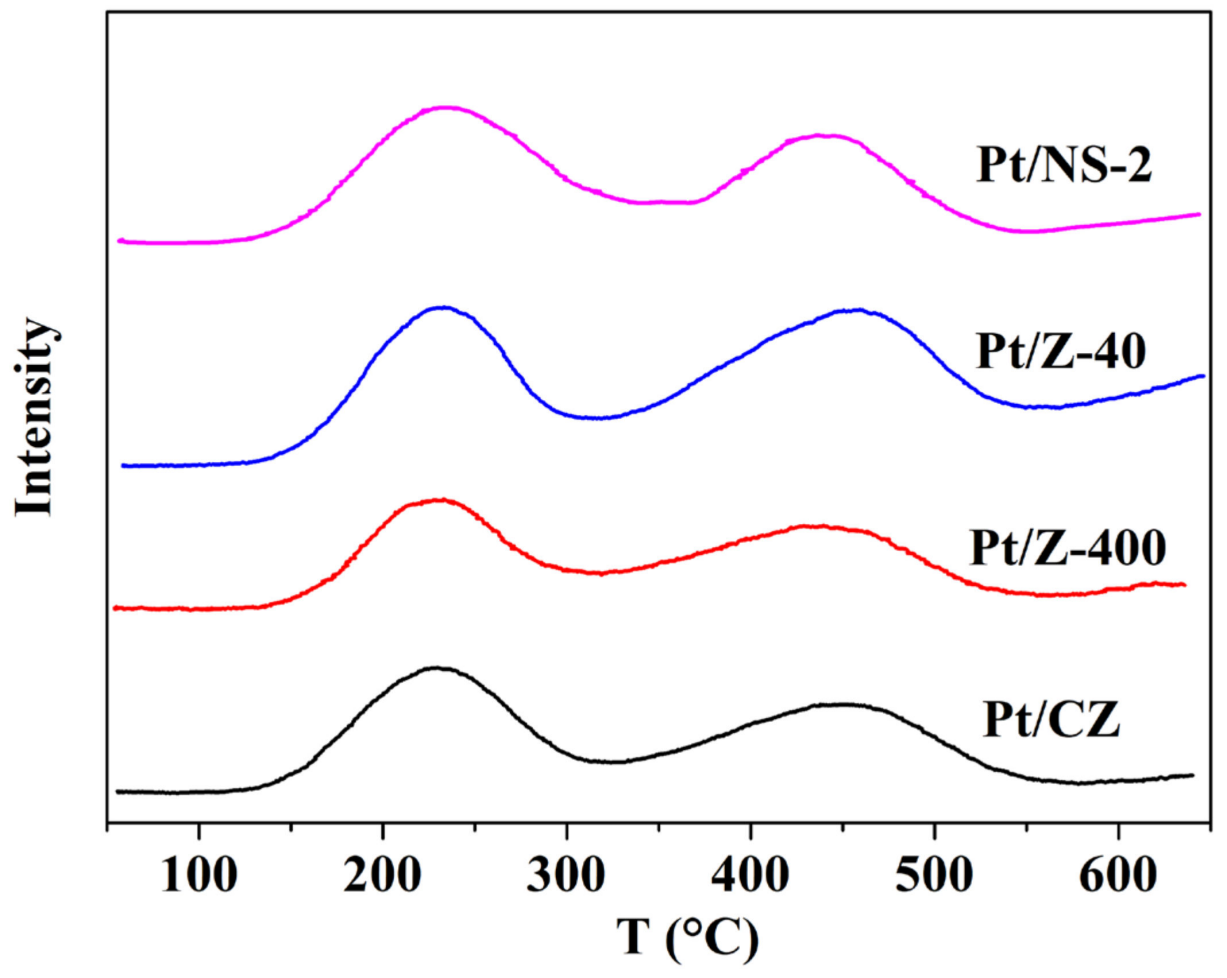
NH_3_–TPD profiles of different Pt-loaded zeolite catalysts.

The NH_3_–TPD profiles of different Pt-loaded zeolite catalysts are displayed in Figure 4. Two NH_3_ desorption peaks are observed for all samples. The low-temperature peak from 100 to 325 °C corresponds to the weak acid adsorption on the catalyst surface, while the peak spanning from 325 to 575 °C is attributed to the strong acid adsorption (detailed acid distribution data are provided in Table 1). Generally, no significant differences in the acid distributions were detected, and the total acidities ranged from 537 µmol/g·NH_3_ to 603 µmol/g·NH_3_. The obtained inductively coupled plasma atomic emission spectroscopy (ICP–AES) spectra showed that the *n*(Si)/*n*(Al) ratios of all samples varied from 46 to 49, suggesting that their crystal sizes did not influence the nucleation of silicon and aluminium species.

The Pt dispersion and particle sizes calculated from the CO chemisorption data are listed in Table 1 (detailed calculation process was in Supporting Information section). The dispersion of Pt particles decreased from 30.3% for Pt/Z-40 to 17.2% for Pt/Z-400 and further to 11.6% for Pt/CZ, suggesting that the hierarchical zeolite with a smaller crystal size facilitated Pt dispersion. The particle sizes calculated from the CO chemisorption data decreased in the order Pt/CZ (9.8 nm) > Pt/Z-400 (6.4 nm) > Pt/Z-40 (3.3 nm). The Pt dispersion on the nanosheet ZSM-5 zeolite surface was slightly lower than that of Pt/Z-40 catalyst with a diameter of approximately 4.2 nm, which could be attributed to the highly exposed surface area of the nanosheet layers, as indicated by the TEM observations.

**Figure 5:**
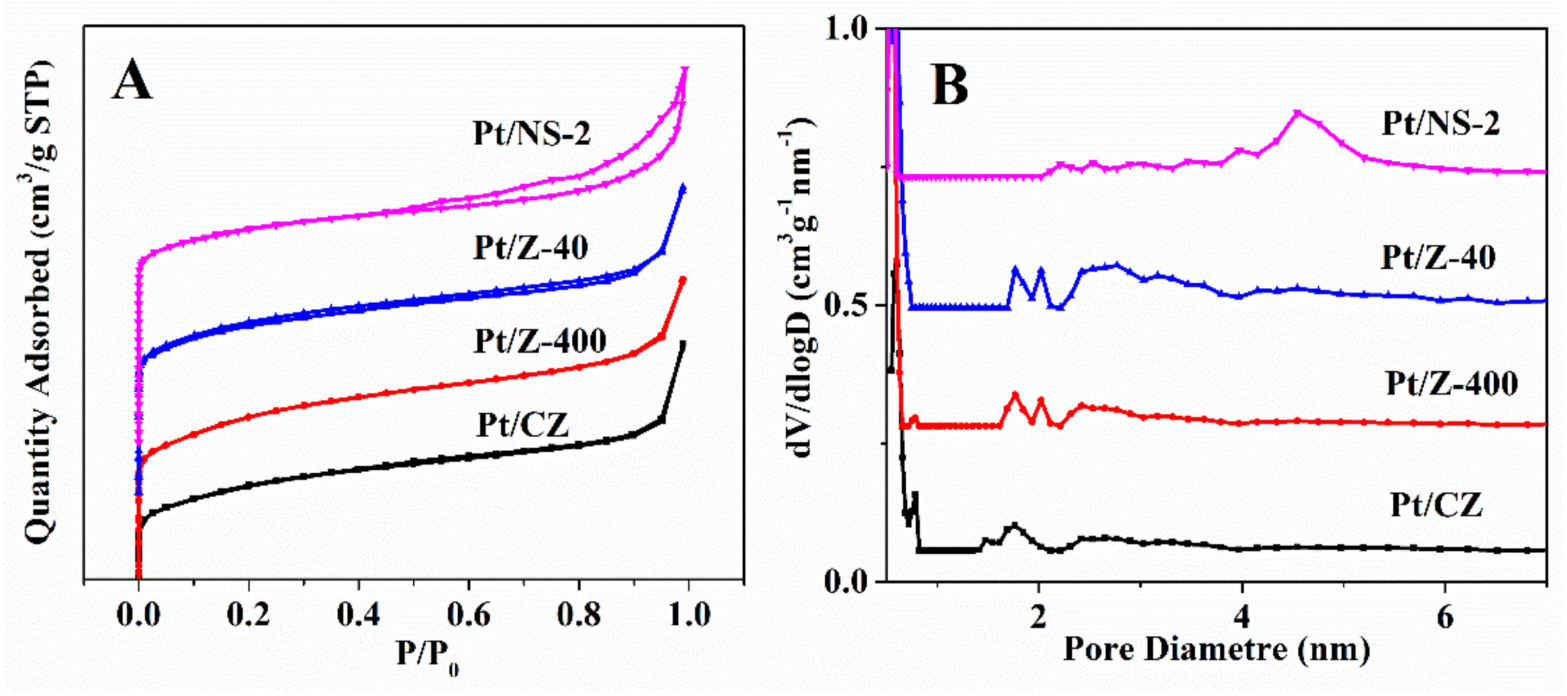
(**A**) N_2_ physisorption isotherms and (**B**) pore size distributions of the Pt-loaded zeolite catalysts.

The N_2_ physisorption isotherms and pore size distributions of the Pt-loaded zeolites are displayed in Figure 5A,B. Here, Pt/CZ, Pt/Z-400, and Pt/Z-40 catalysts show type-I isotherms corresponding to microporous structures. The nanosheet zeolite catalyst (Pt/NS-2) exhibits a type-IV isotherm, indicating the presence of a mesoporous phase. The pore size distributions of the four catalysts calculated by a non-local density functional theory method from the corresponding desorption isotherms significantly differ from each other. All samples contained micropores with diameters of approximately 0.6 nm. After the addition of silicalite-1 seeds, intra-crystalline mesopores with diameters of 2–4 nm were generated between the zeolite nanocrystals (their fraction in Pt/Z-40 catalyst was greater than that in Pt/Z-400 catalyst). The mesopores in the Pt/NS-2 sample with sizes ranging from 2 to 6 nm (mean value: 5 nm) were mainly located in the regions between the adjacent nanosheets and distorted areas inside the thick layers consisting of several nanosheet stacks [35].

The textural properties of the prepared Pt-loaded zeolite catalysts are listed in Table 2. Owing to their hierarchical structures, decreasing the crystal size increased both the total surface areas (*S_BET_*) and mesoporous surface areas (*S_meso_*) of these materials. The lowest *S_BET_* and *S_meso_* values of 365 and 54 m^2^/g were obtained for Pt/CZ catalyst. While for Pt/Z-40, the corresponding magnitudes were 6.2% and 37% higher (414 and 118 m^2^/g, respectively) than that of Pt/Z-400 (390 and 86 m^2^/g, respectively). The nanosheet zeolite sample with a high exposed surface area produced *S_BET_* and *S_meso_* values of 436 and 189 m^2^/g, respectively. In contrast, the micropore surface areas (*S_micro_*) demonstrated the opposite trends (the zeolite catalysts with larger crystal sizes had higher *S_micro_* magnitudes). The measured pore volumes (*V_total_*) strongly correlated with the surface areas. The zeolite catalysts with smaller crystallite sizes exhibited higher mesoporous volumes (*V_meso_*). Thus, the value of 0.282 cm^3^/g obtained for Pt/NS-2 was 3.92, 2.61, and 1.86 times larger than the mesoporous volumes of Pt/CZ, Pt/Z-400, and Pt/Z-40, respectively.

**Figure 6:**
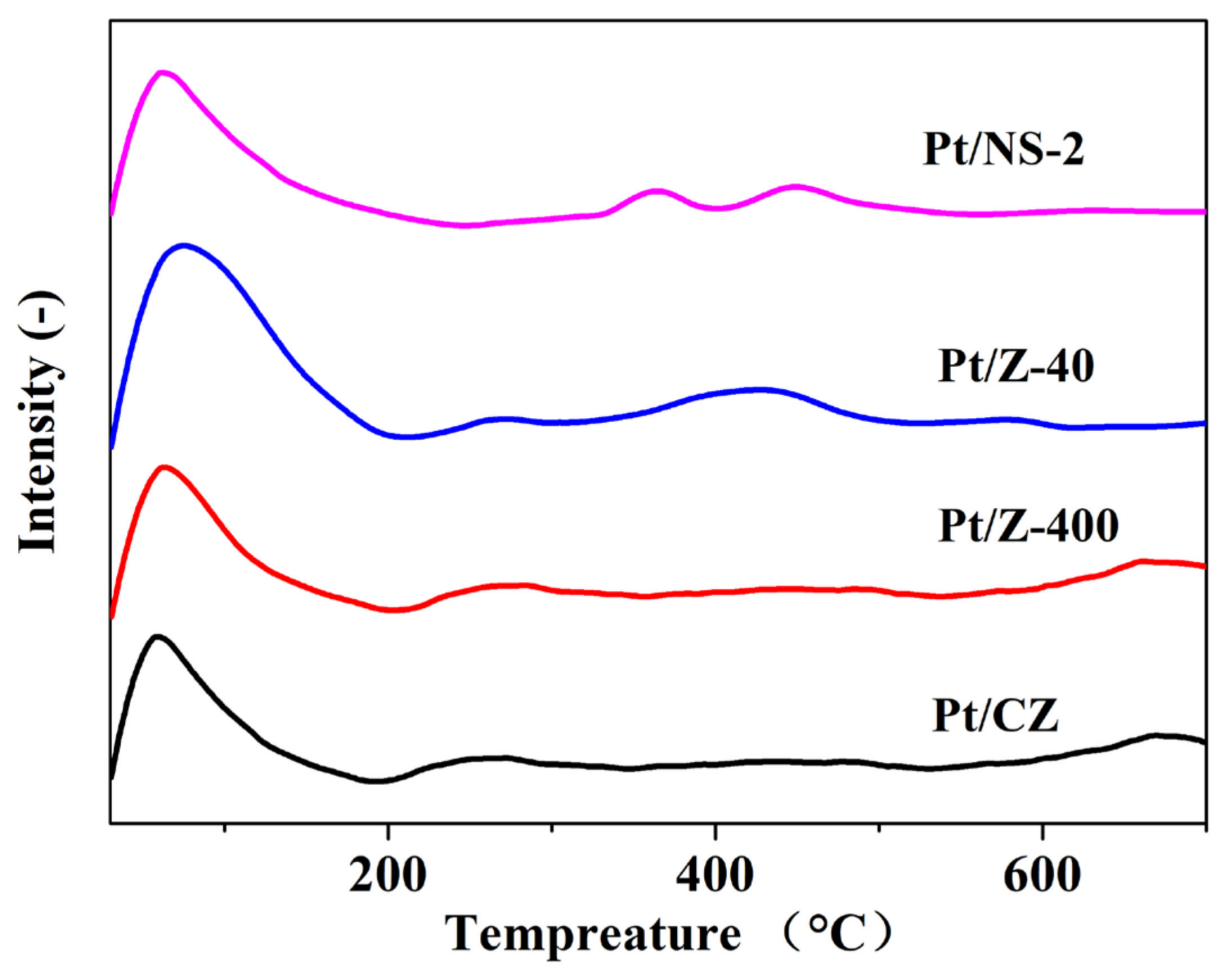
H_2_–TPR profiles of the Pt-loaded zeolite catalysts.

According to the H_2_–TPR profiles presented in Figure 6, all catalyst samples exhibit two reduction peak groups: one below 200 °C and the other one higher than 300 °C. The former peak likely resulted from the reduction of Pt^2+^ ions to Pt^0^ on the catalyst surface [36]. With the increase of temperature to 300 °C under H_2_ atmosphere, the first group Pt^2+^ was all reduced. The second peak group with a higher reduction temperature from 300 to 500 °C suggests the existence of strong interactions between Pt species and the catalyst support [37,38]. With the decrease of zeolite crystal sizes, such H_2_-TPR peaks were detected obviously in Pt/Z-40 and Pt/NS-2 samples, indicating strong interaction between Pt and zeolite surface with decreased zeolite crystal size. Moreover, different from Pt/Z-40, there exist two reduction peak centering at ca. 370 °C and 440 °C in Pt/NS-2, implying more complexed interaction between Pt and exposed nanosheet layers.

**Figure 7:**
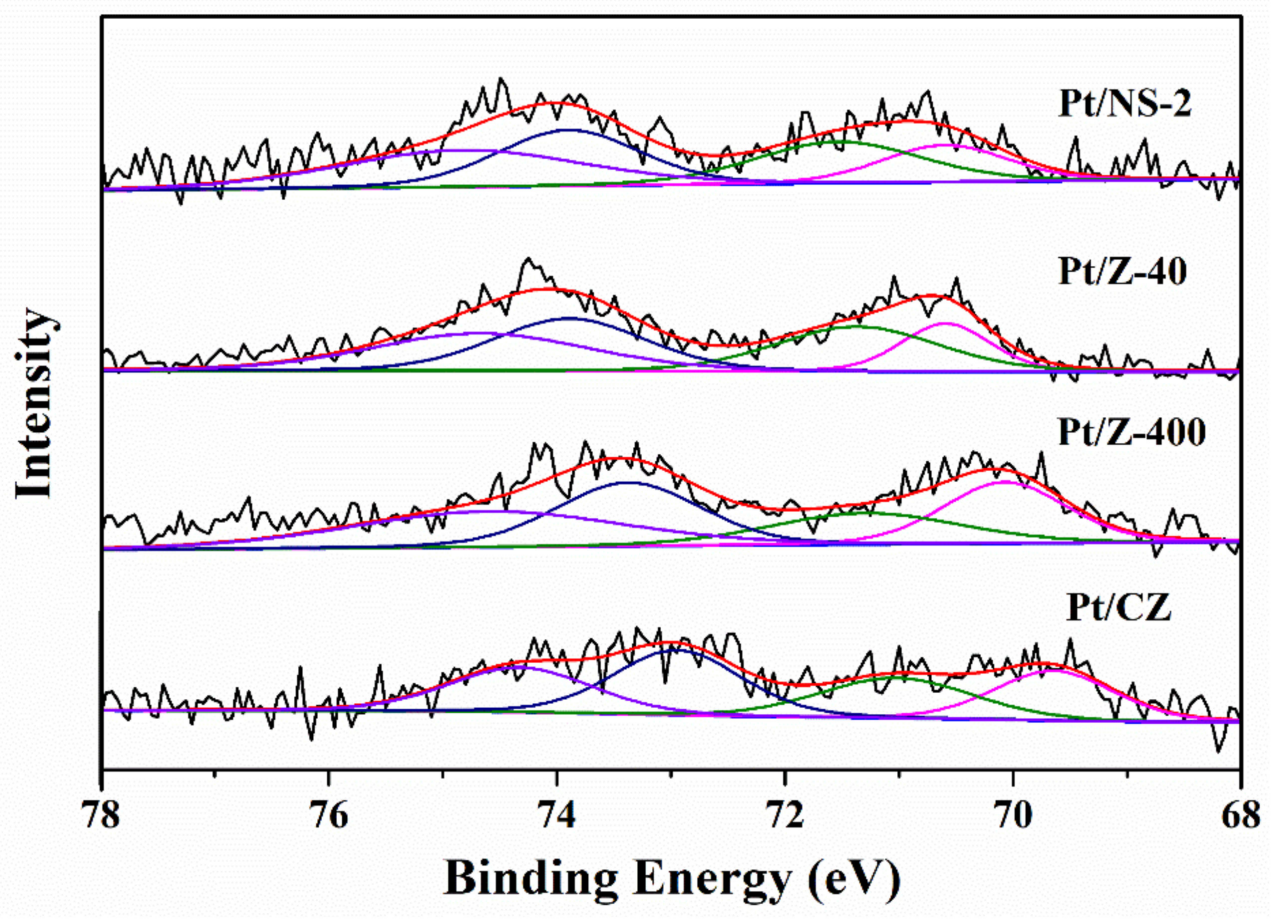
Pt 4f XPS spectra of different Pt-loaded zeolite catalysts.

The XPS spectra of the prepared catalysts are shown in Figure 7. The Al 2p peaks (centred at 73.9 eV) are located in the range of 79–67 eV, which were separated from these spectra [39,40]. The obtained Pt 4f_7/2_ spectra were deconvoluted into two peaks centred at 70.1 and 71.0 eV, and the Pt 4f_5/2_ spectra were deconvoluted into two peaks centred at 73.4 and 74.3 eV as shown in Table 3. The peaks located at 70.1 eV and 73.4 eV originated from Pt^0^ species, and the peaks centred at 71.0 eV and 74.3 eV resulted from Pt^2+^ species [41]. The binding energy of the Pt^0^ (Pt 4f_7/2_) peak increased from 69.6 eV for Pt/CZ to 70.1 eV for Pt/Z-400 and further to 70.6 eV for Pt/Z-40. All Pt 4f peaks exhibited similar trends, indicating that the interactions between Pt atoms and the zeolite carriers were enhanced with a decrease in the zeolite particle size. The XPS spectra of Pt/NS-2 catalyst were similar to those of Pt/Z-40 catalyst. Moreover, the proportion of Pt^0^ species decreased with a decrease in the zeolite crystal size in the order Pt/CZ (53.13%) > Pt/Z-400 (51.50%) > Pt/Z-40 (44.21%) ≈ Pt/NS-2 (44.31%). These results also confirm that the lower carrier sizes and highly dispersed metal particles strengthen the interactions between the metal and the carrier surface, thus decreasing the fraction of Pt^0^ species. Hence, the oxidation state of Pt atoms characterises the interactions of the oxygen framework with Pt nanoparticles, i.e., Pt–zeolite interactions [42].

### 3.2. Catalyst Properties

**Figure 8:**
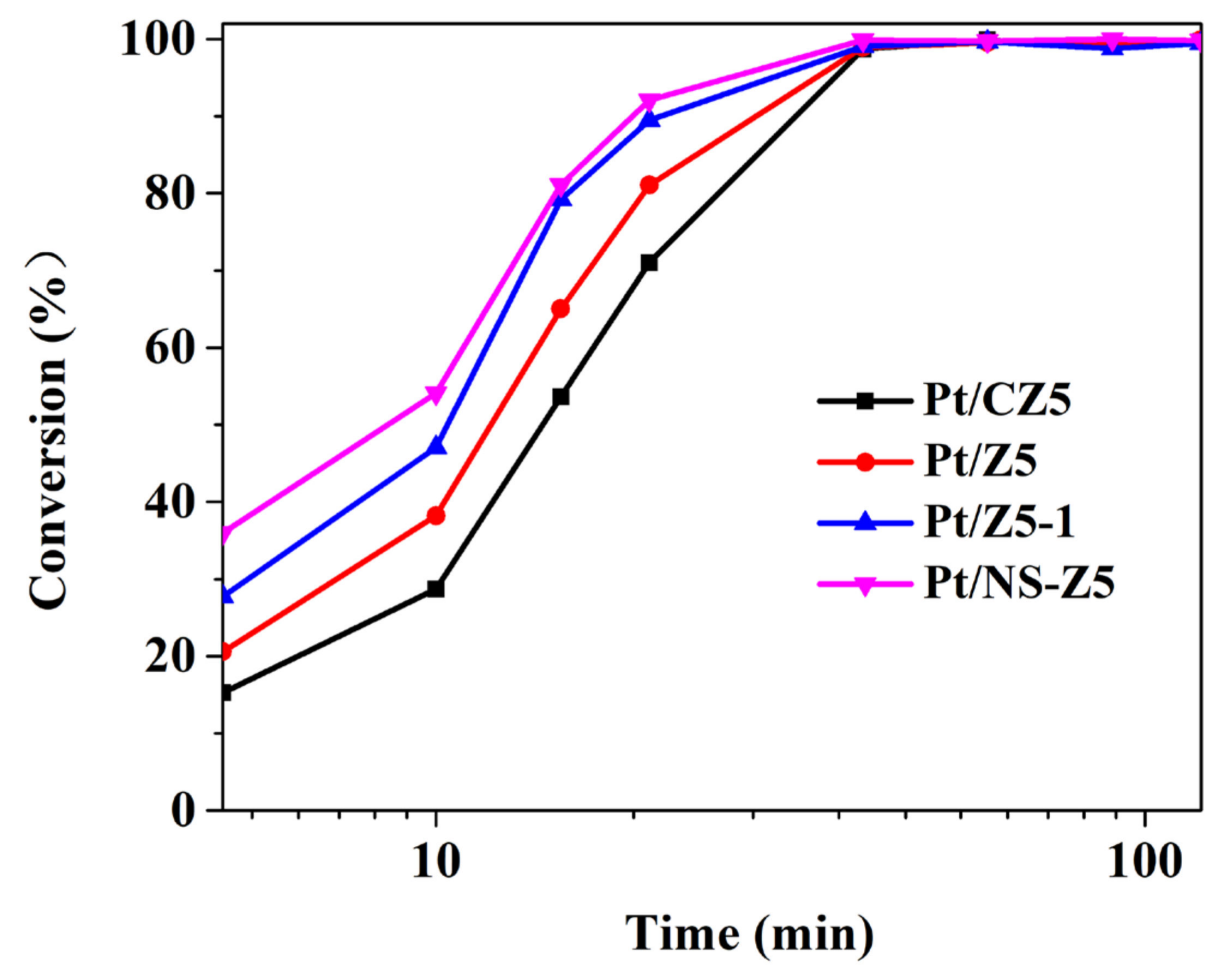
Time-dependent guaiacol conversions obtained for different Pt-loaded zeolite catalysts.

Figure 8 shows the guaiacol conversions plotted as functions of time for different catalysts at a reaction temperature of 250 °C. In general, their values gradually increase with time and reach 100% after 60 min of reaction. Moreover, the catalysts with smaller crystal sizes exhibited higher guaiacol conversions after the same reaction time in the order Pt/NS-2 > Pt/Z-40 > Pt/Z-400 > Pt-CZ, which might be attributed to the faster diffusion of reactant molecules through the mesopores and the resulted highly dispersed Pt nanoparticles. The corresponding product distributions in the liquid phase are displayed in Table 4; here, the selectivities of cyclohexane, phenol, methanol, cyclohexanol, methoxy-cyclohexane, cyclohexanone, 2-methoxycyclohexanol, and 2-methoxycyclohexanone account for more than 99 wt % of the total C yield. Small amounts of anisole, catechol, methylguaiacol, methoxycyclopentane, and benzene were also detected among the obtained products. The selectivity of cyclohexane (the final guaiacol HDO product) exceeded 69 wt % for all the studied catalysts. In particular, at lower crystal sizes, the cyclohexane selectivities in the product mixtures catalyzed by Pt/Z-40 and Pt/NS-2 were approximately 80 wt %.

**Scheme 1:**
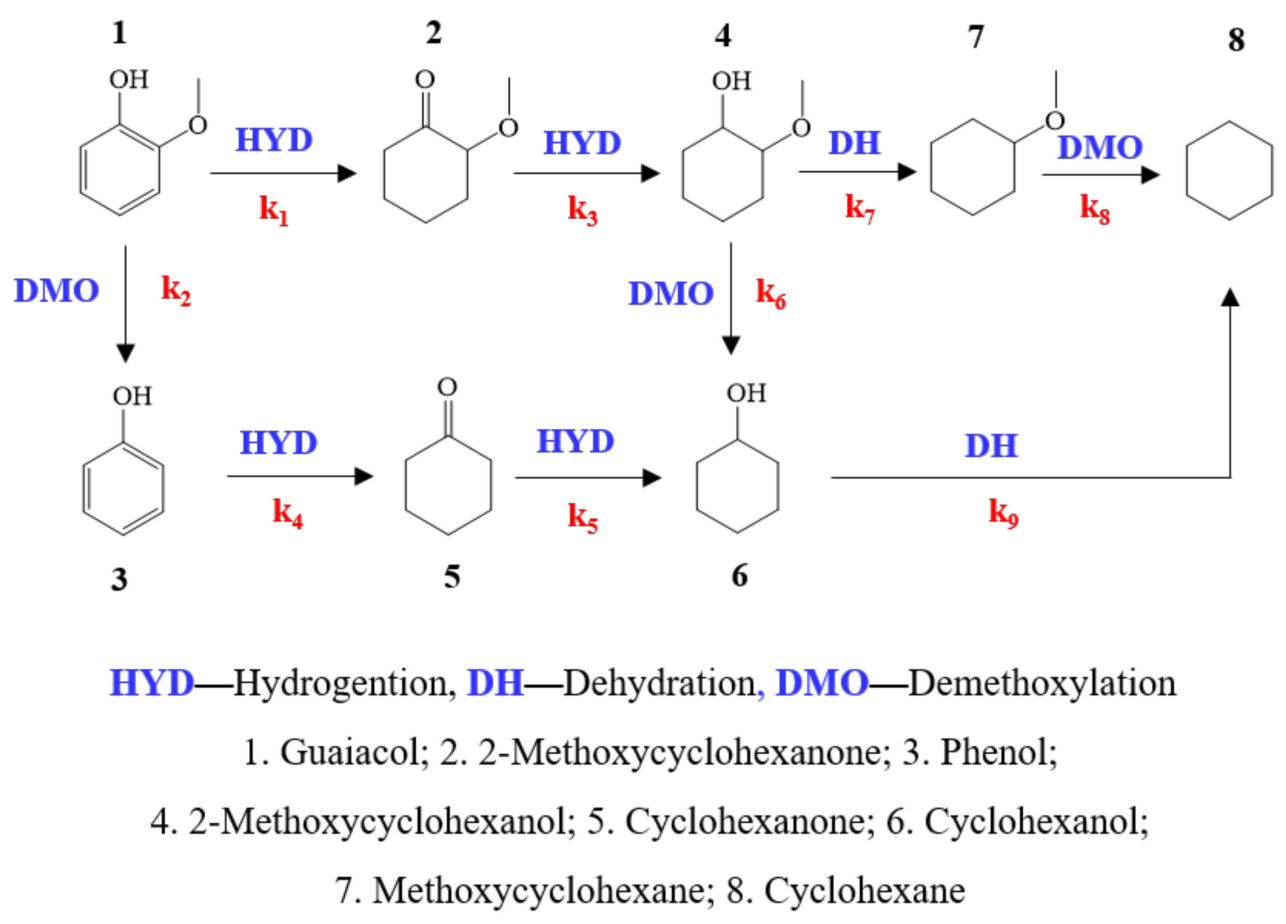
A proposed guaiacol HDO reaction pathway over different Pt-loaded HZSM-5 zeolite catalysts.

From the obtained product distribution and results of the previous work [10,18], we proposed the reaction pathway depicted in Scheme 1. Here, we consider the main products (with C yields in the final mixture greater than 99 wt % except for methanol) as examples. For the catalysts containing both the acidic and metal active sites, guaiacol undergoes a series of hydrogenation (saturation) and hydrogenolysis reactions (including dehydration and demethoxylation), generating various products. First, guaiacol (1#) is transformed into 2-methoxycyclohexanol (2#, 2-methoxycyclohexanone (4#) is an intermediate product) and phenol (3#) though hydrogenation and demethoxylation, respectively. Trace amount of anisole after the guaiacol dehydration (Figure S2) were detected only for Pt/Z-40 and Pt/NS-2 catalysts, which likely resulted from the higher dissociation energy (BDE) of the C(sp_2_)–OH bond (451 kJ/mol, Figure S3A) as compared with that of the C(sp_2_)–OMe bond (387 kJ/mol). In fact, the obtained BDE values indicate that the demethylation route including C(sp_3_)–OAr bond scission is more favorable (201 kJ/mol) than the deoxygenation process. However, almost no catechol species were detected among the reaction products under the specified reaction conditions except for the process conducted over Pt/NS-2 catalyst (0.25 wt %) because the prevalence of the demethoxylation process over demethylation was likely caused by the easy contact between C_aryl_–OMe species and the Pt surface [43], and the nanosheet zeolite had the highest surface area that promoted C_aryl_O–Me adsorption.

Among the primary products, cyclohexanol (6#) was obtained through the hydrogenation of phenol (cyclohexanone (5#) was an intermediate product) and demethoxylation of 2-methoxycyclohexanol (4#). Trace amounts of benzene were detected for Pt/Z-40 (0.08 wt %) and Pt/NS-2 (0.26 wt %) catalysts due to the inhibited deoxygenation of phenol (Figure S2), which was consistent with the results of previous research studies on the HDO of phenol over Ru/HZSM-5 catalyst [44]. The produced 2-methoxycyclohexanol undergoes dehydration in H_2_ atmosphere with the generation of 2-methoxycyclohexane (7#) followed by the formation of cyclohexane (8#) through the demethoxylation of 2-methoxycyclohexane and dehydration of cyclohexanol. Small amounts of methylguaiacol (Figure S2(2)) and methoxycyclopentane (Figure S2(4)) produced by the alkyl transfer reaction were formed over Pt/Z-40 and Pt/NS-2 catalysts, owing to the increase in the catalytic activity of Pt active sites with decreasing zeolite crystal size.

To determine the reaction rate of guaiacol conversion, we measured the product selectivity as a function of the reaction time. It has been shown previously that the HDO of phenolic compounds conducted in the excess of H_2_ represents a first-order reaction with respect to the reactant concentrations [45]. Therefore, the product formation rates were expressed according to the simplified model presented below, where *k_i_* was the reaction rate constant, and *x_i_* was the molar fraction of a product. According to the reaction pathway described in Scheme 1, various reaction rates in the HDO process are characterised by the corresponding rate constants (*k*_1_ to *k*_9_).

$$\frac{dx_{1}}{dt}=-\left(k_{1}+k_{2}\right)x_{1}$$

$$\frac{dx_{2}}{dt}=k_{1}x_{1}-k_{3}x_{2}$$

$$\frac{dx_{3}}{dt}=k_{2}x_{1}-k_{4}x_{3}$$

$$\frac{dx_{4}}{dt}=k_{3}x_{2}-k_{7}x_{4}-k_{6}x_{4}$$

$$\frac{dx_{5}}{dt}=k_{4}x_{3}-k_{5}x_{5}$$

$$\frac{dx_{6}}{dt}=k_{5}x_{5}+k_{6}x_{4}-k_{9}x_{6}$$

$$\frac{dx_{7}}{dt}=k_{7}x_{4}-k_{8}x_{7}$$

$$\frac{dx_{8}}{dt}=k_{8}x_{7}+k_{9}x_{6}$$

*Ki* was calculated via the following procedure. First, the kinetic equations were integrated numerically by a fourth-order Runge–Kutta method. After that, the rate equations were combined with the mass balance equation, and the parameters of the kinetic model were estimated by a least-squares minimization method, in which the following objective function was minimized using the Nelder–Mead simplex search algorithm [46]:

$$O.F.=\frac{\sum_{i}^{all.sample}{\left(x_{i.\exp}-x_{i.calc}\right)}^{2}/x_{i.\exp}}{n_{sample}}\times 100\%$$

where *O.F.* is the objective function, and *x_i.exp_* and *x_i.cal_* are the experimental and calculated concentrations of compound *i*, respectively.

**Figure 9:**
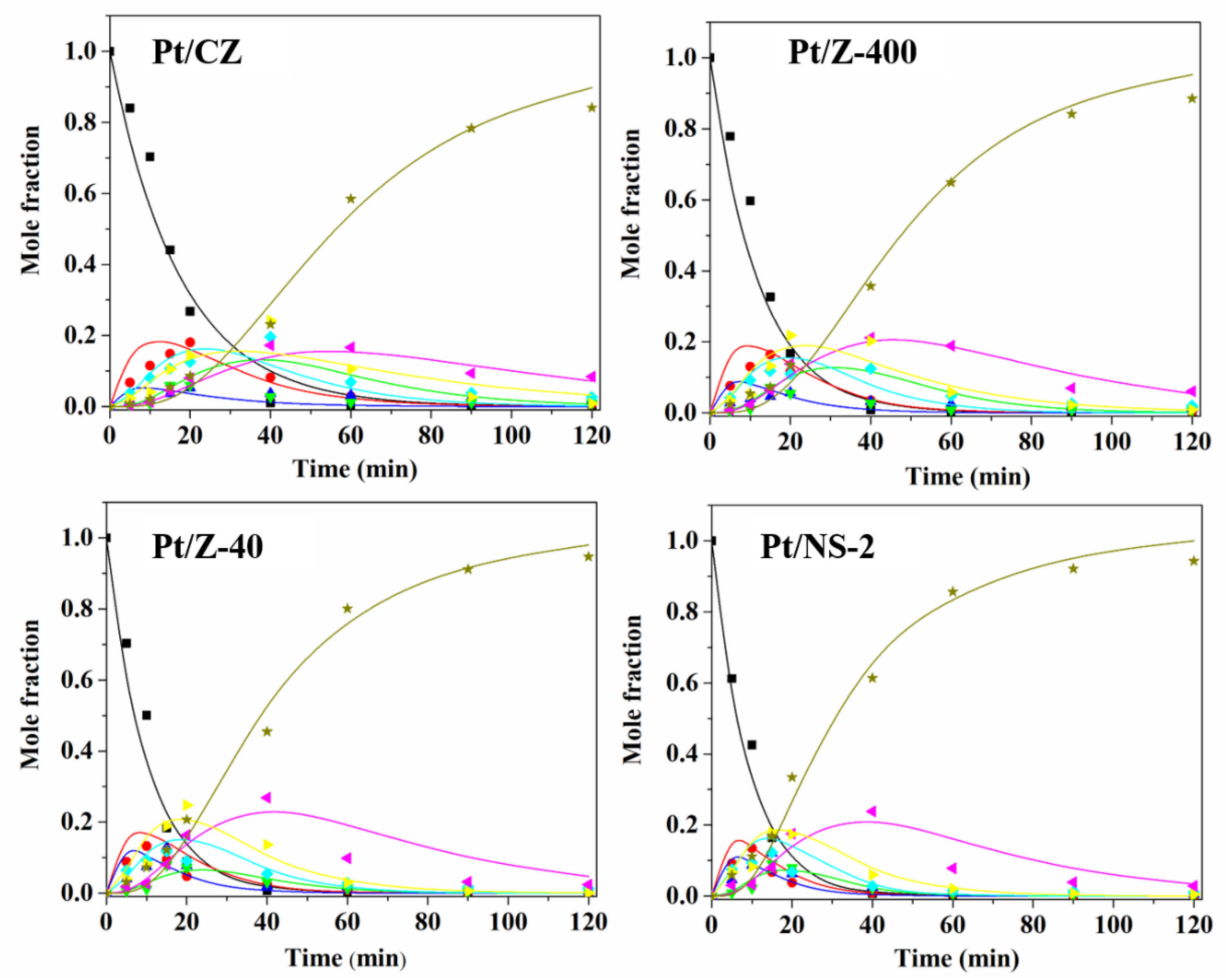
Mole fractions of various products in the reaction mixture obtained at different residence times and temperature of 250 °C. The points denote the experimental data, and the lines represent the calculated values: (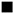) Guaiacol, (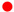) 2-Methoxycyclohexanone, (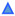) Phenol, (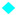) 2-Methoxycyclohexanol, (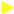) Cyclohexanone, (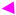) Cyclohexanol, (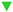) Methoxycyclohexane, (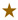) Cyclohexane.

The mole fractions of various reaction products determined at different reaction times are shown in Figure 9 (here, the points represent the experimental values, and the lines denote the calculated value obtained by fitting). According to this figure, the molar fraction of guaiacol decreases gradually with reaction time. At a time of 120 min, almost the entire guaiacol amount has been converted. Meanwhile, the molar fraction of cyclohexane product continues to increase, and the contents of the remaining products first increase and then decrease.

The *k_i_* values calculated for different reaction steps are listed in Table 5. The *k_i_* magnitudes obtained by fitting gradually increased with a decrease in the HZSM-5 zeolite crystal size in the order Pt/NS-2 > Pt/Z-40 > Pt/Z-400 > Pt/CZ. This result is consistent with the surface area and mesopore volumes of the studied catalysts, indicating that the diffusion of reactant molecules is promoted by decreasing the crystal size. Note that the growth rates of the *k_i_* values determined for the same reaction path also exhibited remarkable changes at different catalyst particle sizes. Thus, *k*_1_, *k*_2_, *k*_3_, *k*_6_, and *k_7_* increased faster than *k*_4_, *k*_5_, *k*_8_, and *k*_9_, as shown in Figure S4 and Table S1, because the molecular sizes of guaiacol, 2-methoxycyclohexanol, and 2-methoxycyclohexanone (0.67 nm) were larger than the micropore size of ZSM-5 zeolite (0.55 nm). For this reason, the reaction occurred either in the pore mouth or on the external surface of the micropores [47]. The hierarchical pore structure accelerated the diffusion of hydrocarbon molecules with large volumes. In contrast, monosubstituted cyclohydrocarbons such as phenol, cyclohexanol, and 2-methoxycyclohexane generated during the reaction could be transported through the micropores, leading to the full utilisation of the active sites on the catalyst inner and outer surfaces.

**Figure 10:**
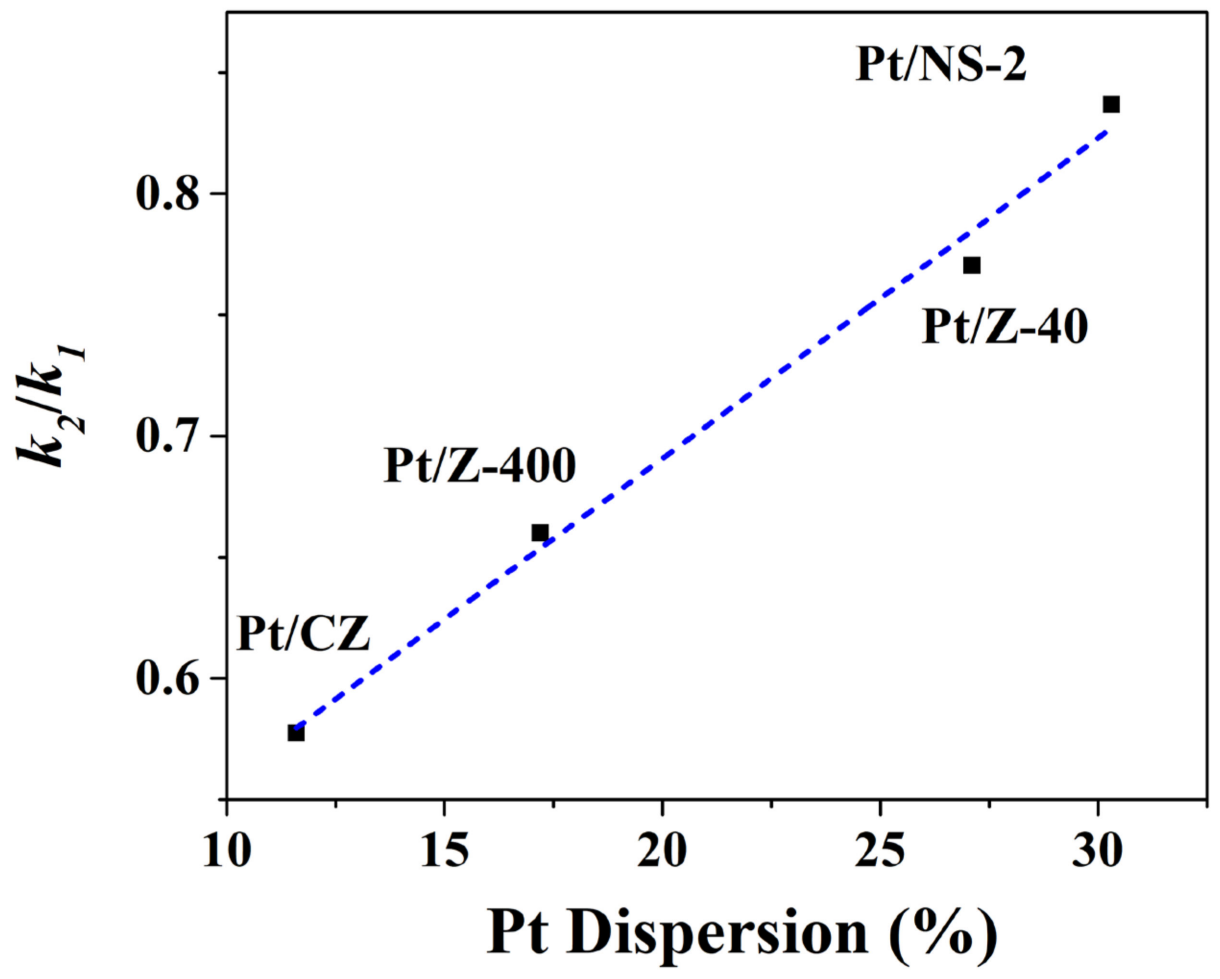
Correlation between the Pt dispersion and the *k*_2_/*k*_1_ ratio.

According to the reaction path diagram, the rate constants of the hydrogenation reaction include *k*_1_, *k*_3_, *k*_4_, and *k*_5_, and those of the deoxygenation reaction consist of *k*_2_, *k*_6_, *k*_7_, *k*_8_, and *k*_9_. In particular, *k*_2_/*k*_1_ is the ratio between the guaiacol deoxygenation and hydrogenation rates. For the metal–acid bifunctional catalysts, it is generally assumed that the hydrogenation reaction occurs on the metal active sites, and the deoxygenation process is related to the acid distribution. Because the total acid contents of different catalysts are very close (as indicated by the NH_3–_TPD results), the kinetics of the deoxygenation reaction may be strongly related to the interactions between the metal and acidic sites. The Pt dispersion exhibits a good linear relationship with *k*_2_/*k*_1_ (Figure 10), indicating that the highly dispersed Pt particles promote the deoxygenation of the reactant. Furthermore, smaller Pt nanoparticles with a highly coordinatively unsaturated surface favor the C−O cleavage through the facile adsorption/stabilisation of −OH/−OCH_3_ species in the transition state, thus facilitating the deoxygenation process [48,49].

When Pt particles are loaded onto the conventional bulky ZSM-5 zeolite, the reactant molecules undergo hydrogenation at the metal active sites and then become deoxygenated to form the final product. Owing to the electron donor effect of the benzene ring, the BED values of C_aryl_–OH/OCH_3_ are significantly higher than those of C_cyclo_–OH/OCH_3_ (Figure S3B). This considerably limits the direct deoxygenation of guaiacol catalyzed by the acidic sites, and the resulting guaiacol hydrogenation rate (*k*_1_) is equal to a half of the rate (*k*_2_) obtained for Pt/CZ catalyst. As the zeolite crystal size decreases, the Pt dispersion increases, and the distance between the metal and acidic sites decreases significantly, which is consistent with the H_2_–TPR and XPS data. The hydrogenation products formed at the metal sites can bind to the adjacent acidic sites more quickly to undergo a deoxygenation reaction. Meanwhile, in the case of a hydrogen spillover on the highly dispersed metal sites, the activated hydrogen species adsorbed on the metal surface can be easily transferred to the adjacent acid sites, promoting the deoxygenation of the hydrocarbons adsorbed on those sites [50]. Therefore, the direct deoxidation rate of guaiacol increases over Pt/Z-40 and Pt/NS-2 catalysts. Some deoxygenation products may also be rapidly transported to the adjacent Pt active sites, leading to the formation of saturated aliphatic compounds through a hydrogenation reaction.

After the decrease in the zeolite crystal size, benzene and methoxycyclopentane products were detected for Pt/Z-40 and Pt/NS-2, further confirming that the deoxygenation and alkyl transfer reactions catalyzed by the acid sites were enhanced by the high surface areas of these catalysts to produce small amounts of methylguaiacol and catechol [51].

## 4. Conclusions

In this study, the HDO of guaiacol was used as a model reaction to explore the influence of Pt/HZSM-5 catalysts with different zeolite particle sizes on the reaction pathway and product selectivity during bio-oil upgrading. In terms of product distribution, the guaiacol conversions of all the prepared catalysts reached values close to 100% after 120 min of the reactions conducted at 250 °C, and the corresponding cyclohexane selectivities were as high as 70 wt %. However, the conversion pathways and the corresponding reaction rates of guaiacol and its intermediates obtained for various catalysts were significantly different for the following reasons: (1) The hierarchical zeolite increased the reaction rate by promoting the diffusion process, especially for bulky molecules such as guaiacol, 2-methoxycyclohexanol and 2-methoxycyclohexanone. (2) The hierarchical HZSM-5 catalyst prepared by the seeding technique (Pt/Z-40 with a thickness of 40 nm) was found to be effective for synthesising highly dispersed Pt-loaded zeolite materials. Therefore, the decreased distance between the Pt active sites and acidic sites strengthened the interactions between the metal species and the catalyst support. The molecules adsorbed onto acidic sites could bind to the metal-activated hydrogen species and were more susceptible to the hydrogenolysis reaction. Therefore, the catalysts containing highly dispersed Pt particles were more suitable for the catalytic deoxygenation of oxygen-containing molecules. (3) Although Pt/Z-40 and Pt/NS-2 catalysts exhibited similar cyclohexane distributions in the final products, the highly exposed external surface of HZSM-5 nanosheets favored more diverse and complex reaction pathways such as those including alkyl transfer and isomerisation processes.

## Figures and Tables

**Table 1:** Acidic properties and Pt distributions of various Pt catalysts.

Sample	n(Si)/n(Al) ^a^	Acid Density (µmol/g·NH_3_)			Pt ^b^ (wt %)	Dispersion ^c^ (%)	Particle Size (nm)	
		W	S	T			CO ^c^	TEM ^d^
Pt/CZ	47	286	251	537	0.95	11.6	9.8	9.6
Pt/Z-400	46	344	241	585	0.90	17.2	6.4	5.9
Pt/Z-40	49	292	311	603	0.92	30.3	3.3	2.8
Pt/NS-2	47	281	272	553	0.88	27.1	4.2	3.9

^a^ Si/Al ratio obtained via ICP–AES analysis. ^b^ Pt loading obtained via ICP–AES analysis. ^c^ Pt dispersion and particle sizes calculated from the pulse CO chemisorption data. ^d^ Mean particle size obtained by measuring more than 150 particles in the TEM image.

**Table 2:** Textural properties of different Pt-loaded zeolite catalysts.

Samples	Specific Surface Area (m^2^/g)			Pore Volume (cm^3^/g)		
	*S_BET_* ^a^	*S_micro_* ^b^	*S_meso_* ^b^	*V_total_* ^c^	*V_micro_* ^d^	*V_meso_* ^e^
Pt/CZ	365	311	54	0.265	0.193	0.072
Pt/Z-400	390	304	86	0.336	0. 228	0.108
Pt/Z-40	414	296	118	0.392	0.240	0.152
Pt/NS-2	436	247	189	0.473	0.191	0.282

^a^*S_BET_* is the Brunauer–Emmett–Teller (BET) surface area obtained from the N_2_ adsorption isotherm in a relative pressure range (*P*/*P_0_*) of 0.05–0.20. ^b^ *S_micro_* and *S_meso_* are the micropore and mesopore surface areas, respectively, determined by the t-plot method. ^c^ *V_total_* is the total pore volume calculated from the N_2_ uptake at a relative pressure (*P*/*P_0_*) of 0.99. ^d^ *V_micro_* is the micropore volume calculated by the t-plot method. ^e^ *V_meso_* is the mesopore volume calculated by the formula *V_meso_* = *V_t_* − *V_micro_*.

**Table 3:** XPS parameters of different Pt-loaded zeolite catalysts.

Catalysts	Pt 4f_7/2_	Pt 4f_5/2_	Origin	Ratio (%)
	BE ^a^ (eV)	BE (eV)		
Pt/CZ	69.6	72.9	Pt^0^	53.13
	71.0	74.3	Pt^2+^	46.87
Pt/Z-400	70.1	73.4	Pt^0^	51.50
	71.3	74.6	Pt^2+^	48.50
Pt/Z-40	70.6	73.9	Pt^0^	44.21
	71.4	74.7	Pt^2+^	55.79
Pt/NS-2	70.6	73.9	Pt^0^	44.31
	71.5	74.8	Pt^2+^	55.69

^a^ BE is the binding energy.

**Table 4:** Mass fractions of various products obtained during the hydrodeoxygenation (HDO) of guaiacol over different Pt-loaded catalysts ^a^.

Catalysts	Pt/CZ	Pt/Z-400	Pt/Z-40	Pt/NS-2
Product	Mass Fractions (wt %)			
Cyclohexane	69.15	73.62	80.97	79.43
Methoxycyclohexane	1.11	0.81	0.39	0.42
Cyclohexanol	8.88	6.53	2.63	3.99
Cyclohexanone	0.84	0.87	0.30	0.44
2-Methoxycyclohexanol	3.36	2.62	1.75	1.33
2-Methoxycyclohexanone	0.92	0.48	0.30	0.26
Phenol	1.76	1.18	0.60	0.55
Methanol	13.99	13.88	12.76	12.66
Benzene	0.00	0.00	0.08	0.26
Methoxycyclopentane	0.00	0.00	0.17	0.18
Anisole	0.00	0.00	0.04	0.07
Methylguaiacol	0.00	0.00	0.00	0.16
Catechol	0.00	0.00	0.00	0.25

^a^ Reaction conditions: temperature of 250 °C, *t* = 120 min.

**Table 5:** Calculated rate constants for the HDO of guaiacol.

Sample	Pt/CZ	Pt/Z-400	Pt/Z-40	Pt/NS-2
*k*_1_ (×10^−4^)	6.58	7.13	7.91	12.2
*k*_2_ (×10^−4^)	3.80	4.71	6.62	9.42
*k*_3_ (×10^−4^)	12.40	13.20	15.34	21.38
*k*_4_ (×10^−4^)	21.30	23.50	24.76	26.91
*k*_5_ (×10^−4^)	8.53	10.60	11.60	13.48
*k*_6_ (×10^−4^)	3.62	4.11	6.43	9.37
*k*_7_ (×10^−4^)	7.62	8.20	10.71	15.88
*k*_8_ (×10^−4^)	9.16	10.5	12.29	13.72
*k*_9_ (×10^−4^)	6.81	8.23	9.57	10.40

## Supplementary Materials

The following are available online at https://www.mdpi.com/2079-4991/10/11/2246/s1, 1.1: Materials, 1.2: Synthesis of nanosheets HZSM-5, 1.3: Synthesis of bulky HZSM-5, 2.1. Calculation of Pt dispersion, Figure S1. XRD patterns (A), size distributions (B) and TEM images (C) of silicalite-1 nanocrystal seeds, Figure S2. Reaction pathway of guaiacol and its-intermediates, Figure S3. The molecular schematic diagram of (A) guaiacol and (B) 2-Methoxycyclohexanol, Figure S4. The increasing rate of ki under different prepared Pt loaded HZSM-5 zeolite catalysts.
