# Supplementary material for: Effects of Crystallite Sizes of Pt/HZSM-5 Zeolite Catalysts on the Hydrodeoxygenation of Guaiacol
Source: Nanomaterials (Basel). 2020 Nov 12;10(11):2246. doi: 10.3390/nano10112246 (PMC7698081; doi:10.3390/nano10112246)
Supplement: Supplementary file 1 [file nanomaterials-10-02246-s001.pdf]

nanomaterials

Supplementary Material

Effects of Crystallite Sizes of Pt/HZSM-5 Zeolite Catalysts on the Hydrodeoxygenation of Guaiacol

Haonan Duan [1], Yajie Tian [1,*], Siyuan Gong [2], Bofeng Zhang [2], Zongjing Lu [2], Yinqiang Xia [3,*], Yawei Shi [4] and Congzhen Qiao [1,*]

[1] Henan Province Engineering Research Center of Catalytic Reaction, College of Chemistry and Chemical Engineering, Henan University, Kaifeng 474004, China; dhn377143955@163.com (H.D)
[2] Key Laboratory for Green Chemical Technology of Ministry of Education, School of Chemical Engineering and Technology, Tianjin University, Tianjin 300072, China; gsygsy@tju.edu.cn (S.G.); zhangbofeng@tju.edu.cn (B.Z.); zongjinglu@tju.edu.cn (Z.L.)
[3] College of Food Science and Engineering, Northwest A&F University, Yangling 712100, China
[4] College of Environmental Science and Engineering, Dalian Maritime University, Dalian 116026, China; shiyawei@tju.edu.cn
* Correspondence: yjtian@henu.edu.cn (Y.T.); xiatengyu@163.com (Y.X.); qiaocongzhen@henu.edu.cn (C.Q.); Tel.: +86-371-2388-1589 (Y.T.)

1.1. Materials

Aluminum sulfate hydrate ($Al_2(SO_4)_3·18H_2O$, ≥ 98.0), tetraethyl orthosilicate (TEOS, ≥ 98%), ammonium nitrate (NH_4NO_3, ≥ 99.0%), sodium hydroxide (NaOH, ≥ 99.0%), sulfuric acid (H_2SO_4, 95.0–98.0%) were supplied by Guangfu Chemical Reagent Company (Tianjin, China). 1-Bromodocosane (≥ 98.0%), diethyl ether (DEE, ≥ 99.0) and 1-bromohexane (≥ 98%) were supplied by Sigma-Aldrich. N, N, N′, N′-tetramethyl-1,6-diaminohexane (≥ 98%), tetrapropylammonium hydroxide (TPAOH, 25% in H_2O), acetonitrile (HPLC grade) and toluene (≥ 99.0%) were purchased from *J&K*.

1.2. Synthesis of Nanosheets HZSM-5

The polyquaternary ammonium surfactant was synthesized following a reported procedure as follows: 18.5 g of 1-bromodocosane and 86g of N, N, N′, N′-tetramethyl-1,6-diaminohexane were dissolved in 500 mL of acetonitrile/toluene mixture (1:1, v/v) in the glove box. The mixture was then transferred out of the glove box and reacted at 70 °C under magnetic stirring for 10 h. After cooling to ambient temperature, the precipitated product was filtered, washed with DEE, and dried in a vacuum oven at 70 °C overnight. 28 g of the resultant product and 12.5 g of 1-bromohexane were dissolved in 150 mL of acetonitrile in the glove box and then refluxed at 85 °C under magnetic stirring (500 rpm) for 10 h in a fume hood. The obtained product was the surfactant $[C_{22}H_{45}N^+(CH_3)_2C_6H_{12}N^+(CH_3)_2C_6H_{13}](Br^{-1})_2$, designated as C_{22-6-6}.

The recipe used for synthesis of nanosheet HZSM-5 zeolites was as follows: $30Na_2O/Al_2O_3/100SiO_2/10C_{22-6-6}/4000H_2O/18H_2SO_4$. Typically, the hydrothermal synthesis was performed by dissolving 0.7g NaOH in 3.0682 g DI water, dissolving 0.4235 g H_2SO_4 in 4.4471 g DI water, and subsequently adding the basic solution dropwise to the acidic solution under vigorous stirring. After cooling to ambient temperature, a desired amount of $Al_2(SO)_4·18H_2O$ was dissolved in the mixture. TEOS was then added to the mixture and the mixture was stirred vigorously at room temperature for 20 h using a magnetic stirrer. Finally, the mixture was mixed with a C_{22-6-6} solution which was prepared by dissolving 2.1163 g C_{22-6-6} in 13.476 g DI water at 60 °C. After continuously stirring for 4 h at room temperature, the resultant gel was transferred into a Teflon-lined stainless-steel autoclave, followed by crystallization for 5 days in rotating at 150 °C. After crystallization, the zeolite product was filtered, washed with DI water, and dried at 120 °C overnight. All samples were calcined in dry air at 550 °C (increasing at 1 °C/min) for 6 h.

1.3. Synthesis of Bulky HZSM-5

The starting mixture had a molar composition of $1Al_2O_3$: $100SiO_2$: 40TPAOH: $3000H_2O$. Tetraethylorthosilicate (TEOS, 98%) was used as the silica source, and aluminum sulfate (Al2(SO4)3·18H2O, 98%) was used as the alumina source. The crystallization was conducted at 170 °C for 48 h in a teflon-lined stainless steel autoclave under rotation (120 r/min). The solid product was recovered by centrifugation and washed with deionized water, until the mother liquid showed a pH value of 7–8, followed by drying at 110 °C overnight and calcination at 550 °C for 8 h in air.

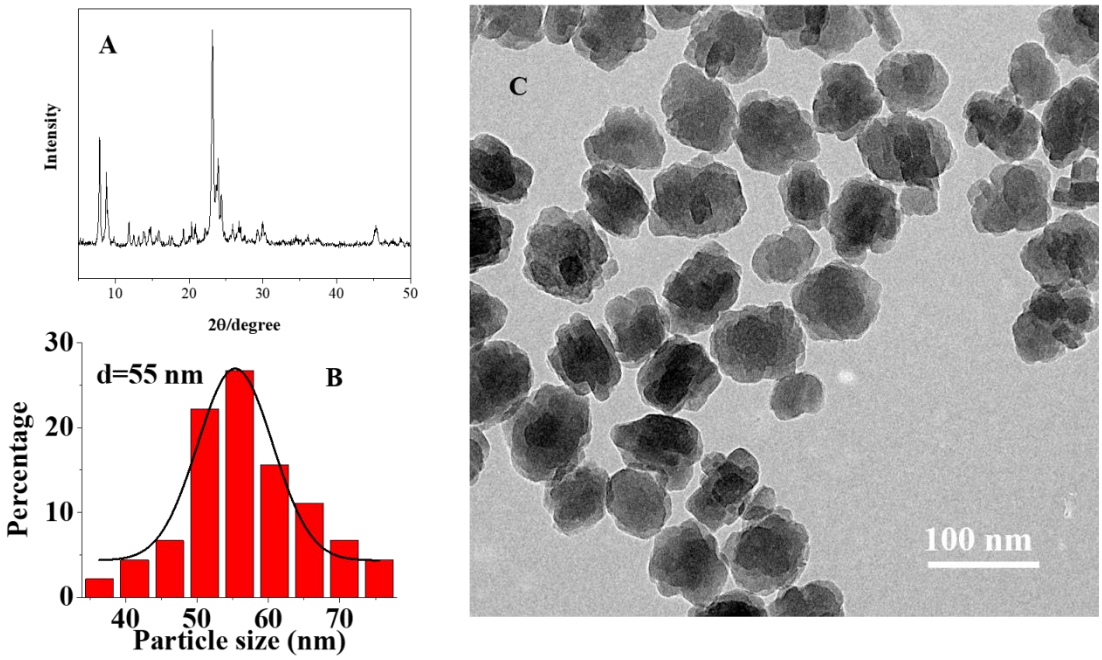

Figure S1. XRD patterns (**A**), size distributions (**B**) and TEM images (**C**) of silicalite-1 nanocrystal seeds.

2.1. Calculation of Pt Dispersion

The dispersion of Pt nanoparticles (*D*%) was calculated as:

$$D(\%) = \frac{n \times M_{Pt}}{X \times Y}$$

where n is the adsorbed amount of CO per gram of catalyst. M_{Pt} is the molar mass of Pt. X is the Pt loading and Y is deemed as 1, assuming an adsorption of one CO molecule per Pt atom. The average Pt particle size (P) was estimated as:

$$P = \frac{1}{\rho \times S \times D}$$

where ρ is the density of Pt nanoparticles. S represents the maximum surface area of Pt nanoparticles and D stands for the metal dispersion. All these results were calculated by the AMI-300 Catalyst Characterization System (Altamira Instruments).

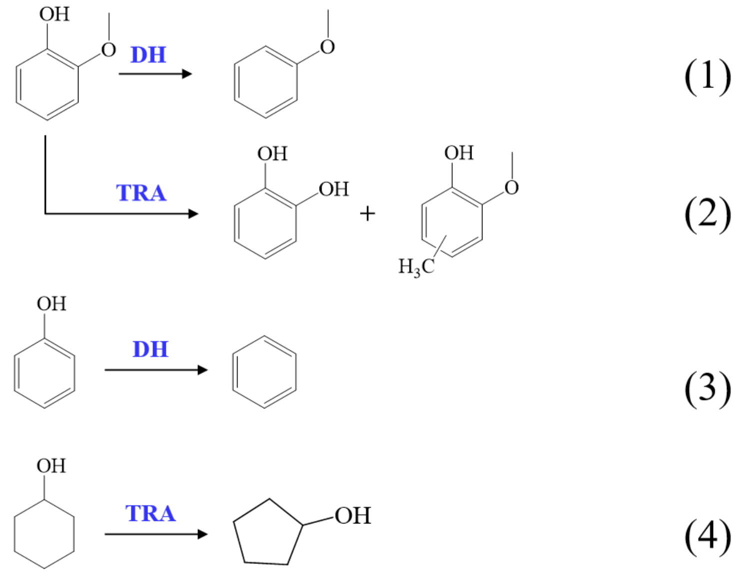

Figure S2. Reaction pathway of guaiacol and its-intermediates.

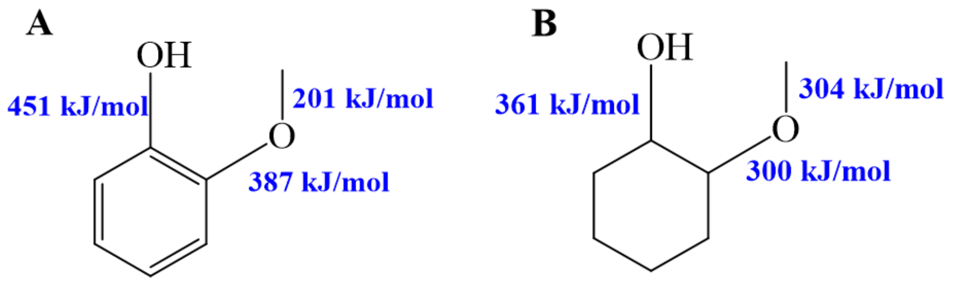

Figure S3. The molecular schematic diagram of (**A**) guaiacol and (**B**) 2-Methoxycyclohexanol. Bond dissociation energies (BDEs, KJ/mol) of C-O bands ca;cu;ated at B3LYP/6-311G (d, p) level.

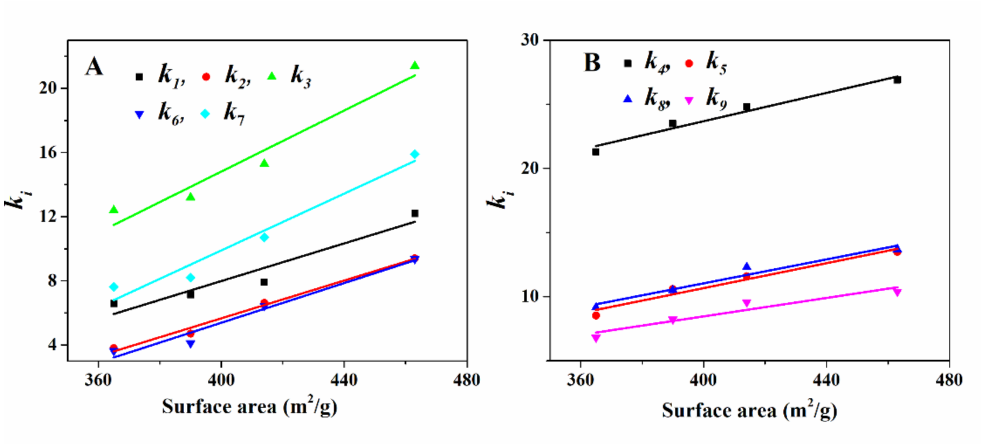

Figure S4. The increasing rate of k_i under different prepared Pt loaded HZSM-5 zeolite catalysts: (**A**) k_1, k_2, k_3, k_6, k_7; (**B**) k_4, k_5, k_8, k_9.

Table S1. The increasing rate of k_i under different prepared Pt loaded HZSM-5 zeolite catalysts.

k_i	k_1	k_2	k_3	k_4	k_5	k_6	k_7	k_8	k_9
k_i'	0.058	0.059	0.095	0.054	0.048	0.062	0.088	0.046	0.036
